# Structural Characterization of *de Novo* Designed L_5_K_5_W Model Peptide Isomers with Potent Antimicrobial and Varied Hemolytic Activities

**DOI:** 10.3390/molecules18010859

**Published:** 2013-01-11

**Authors:** Seo-Jin Kim, Jae-Seok Kim, Yoo-Sup Lee, Dae-Won Sim, Sung-Hee Lee, Young-Yil Bahk, Kwang-Ho Lee, Eun-Hee Kim, Sung-Jean Park, Bong-Jin Lee, Hyung-Sik Won

**Affiliations:** 1 Department of Biotechnology, Research Institute for Biomedical and Health Science, College of Biomedical and Health Science, Konkuk University, Chungju, Chungbuk 380-701, Korea; 2 Division of Magnetic Resonance, Korea Basic Science Institute, Ochang, Chungbuk 363-883, Korea; 3 College of Pharmacy, Gachon University, 534-2 Yeonsu 3-dong, Yeonsu-gu, Incheon 406-799, Korea; 4 Research Institute of Pharmaceutical Sciences, College of Pharmacy, Seoul National University, Seoul 151-742, Korea

**Keywords:** amphipathic helical peptides, antimicrobial peptides, *de novo* design, hemolytic activity, L_5_K_5_W isomers, structure-activity relationships, tryptophan

## Abstract

In an effort to develop short antimicrobial peptides with simple amino acid compositions, we generated a series of undecapeptide isomers having the L_5_K_5_W formula. Amino acid sequences were designed to be perfectly amphipathic when folded into a helical conformation by converging leucines onto one side and lysines onto the other side of the helical axis. The single tryptophans, whose positions were varied in the primary structures, were located commonly at the critical amphipathic interface in the helical wheel projection. Helical conformations and the tryptophanyl environments of the 11 L_5_K_5_W peptides were confirmed and characterized by circular dichroism, fluorescence and nuclear magnetic resonance spectroscopy. All of the isomers exhibited a potent, broad-spectrum of antibacterial activity with just a slight variance in individual potency, whereas their hemolytic activities against human erythrocytes were significantly diversified. Interestingly, helical dispositions and fluorescence blue shifts of the peptides in aqueous trifluoroethanol solutions, rather than in detergent micelles, showed a marked linear correlation with their hemolytic potency. These results demonstrate that our *de novo* design strategy for amphipathic helical model peptides is effective for developing novel antimicrobial peptides and their hemolytic activities can be estimated in correlation with structural parameters.

## Abbreviations

AMP: antimicrobial peptide
CD: circular dichroism
CMC: critical micelle concentration
COSY: correlated spectroscopy
DPC: dodecylphosphocholine
GM: geometric mean of the MICs
MHC: minimal hemolytic concentration
MIC: minimal inhibitory concentration
[θ]: mean residue molar ellipticity
NATA: *N*-acetyl-L-tryptophanamide
PB: phosphate buffer
SDS: sodium dodecylsulfate
TFE: trifluoroethanol
TI': pseudo-therapeutic index

## 1. Introduction

Antimicrobial peptides (AMPs), defined as genetically encoded, endogenous antibiotic peptides, have evolved during the host-pathogen relationship in most living organisms [1,2,3,4,5]. Considering their pleiotrophic functions in diverse cellular processes such as inflammation, angiogenesis, and wound healing, as well as the antibiotic response, AMPs are now being redefined as “host defense peptides” constituting a ubiquitous, integral part of innate immunity [1,2]. The physiological functions of AMPs are also associated with human health and diseases, including intestinal homeostasis, pregnancy maintenance, psoriasis, atopic dermatitis, rosacea, periodontal disease, brain disease, *etc.* [1,5,6,7,8,9,10]. AMPs do not merely act as direct microbicidal agents but also exhibit additional antimicrobial action by the dissolution and suppression of biofilm formation and the promotion of phagocytosis [2]. Other useful activities, such as anti-inflammation, anti-tumor, and insulinotropic activities, have been also reported for certain AMPs [11,12,13,14,15,16]. Consequently, AMPs, as promising therapeutic agents, are being applied to the clinical and commercial development and some have reached clinical trials [2]. At present, a majority of the most improved AMPs for clinical application target anti-infectious diseases due to their desirable features, such as a broad-spectrum microbicidal activity, which includes Gram-positive and -negative bacteria, fungi, parasites, and viruses, rapid onset of killing microbes, and a low propensity for developing resistance [17,18,19,20]. In particular, AMPs are receiving great attention as promising alternatives to conventional antibiotics to combat antibiotic-resistant bacteria [2,3,19]. However, in terms of clinical and industrial applications, there are some drawbacks of AMPs, including high production costs, poor pharmacokinetic properties, *etc.* [5,17,18,19,20]. To address these limitations, we have attempted to develop small size antimicrobial peptides with a simple amino acid composition [21,22], which would be more favorable as lead molecules to reduce production costs and to facilitate pharmaceutical optimization [2,20,21]. To generate such peptides, in the present study, we designed a series of cationic model peptides capable of folding into amphipathic α-helical conformations.

**Figure 1:**
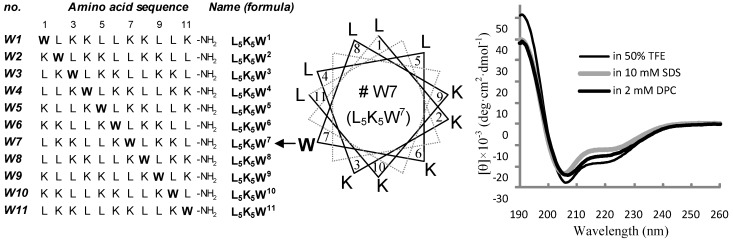
Peptide design and conformational validation. Amino acid sequence of each L_5_K_5_W^n^ model peptide (left panel) is labeled with the serial number on the left and the peptide name as a formula on the right. An example of the helical wheel diagram (middle panel) is depicted for the W7 peptide. Far-UV CD spectra (right panel) of the W7 peptide were measured in 10 mM phosphate buffer (pH 6.5) containing 50% TFE, 10 mM SDS micelles and 2 mM DPC micelles, respectively.

Cationic, amphipathic α-helical peptides constitute a representative class of AMPs that are widely distributed and the most well established in structure and mode of action [23,24,25,26]. Their positive charges are thought to be critical for selectivity between the anionic bacterial membrane surface and the zwitterionic human membrane surface, while the amphipathic helix is a prerequisite conformation for membrane permeation by the peptides. Through prior investigations on the amphibian antimicrobial peptides, gaegurins [27], and their peptide analogues, we found that tryptophanyl substitutions at certain positions in inactive fragments of amphipathic helical AMPs could confer antimicrobial activity [28,29,30,31]. Based on these findings, we have established a simple strategy to design amphipathic helical AMPs with a relatively short length and a simple amino acid composition [21,22]. In this scheme, leucines and lysines are used to impart amphipathic helical properties and a single tryptophan is positioned at the critical amphipathic interface between the end of the hydrophilic (lysyl) side and the start of hydrophobic (leucyl) side, in the helical wheel projection (refer to Figure 1). Some 7- to 13-residue peptides generated under that rule have indeed exhibited antimicrobial activity and one of the most potent peptides was determined to be the L_5_K_5_W^6^ peptide (sequence: KKLLKWLKKLL-amide) [22]. In the previous design, the lone tryptophan was located in the middle of the primary sequence. To further investigate the effect of the tryptophanyl position and to develop improved molecules, we tested 11 peptide isomers having the L_5_K_5_W formula, and their structure-activity relationships are reported in this paper.

## 2. Results and Discussion

### 2.1. Peptide Design and Conformational Validation

Amino acid sequences of the tested L_5_K_5_W^n^ model peptides are summarized in Figure 1. Three kinds of amino acids were used for the design of sequence to generate peptides with simple amino acid compositions. The hydrophobic amino acid leucine and the positively-charged amino acid lysine are known to have a strong helix-forming potential [32,33], and are frequently found in natural amphipathic helical AMPs. The amphiphilic amino acid tryptophan was chosen for its prominent role in membrane interaction of proteins and peptides, by serving as an float on the membrane [28,30,34,35]. According to the *de novo* design scheme created previously [21,22], all of the peptide sequences were devised to be perfectly amphipathic when folded into α-helical structures, by converging five leucines onto one side and five lysines onto the other side of the helical axis. The single tryptophan, of which position in the primary structure was varied in individual peptides, was always located at the critical amphipathic interface between the hydrophilic (lysyl) ending side and the hydrophobic (leucyl) starting side, as illustrated in the helical wheel diagram. As an example, the helical wheel projection of the W7 (L_5_K_5_W^7^) peptide, which showed the most favorable activity (refer to the following section), is presented in Figure 1. The peptides are commonly positively charged due to the presence of the lysines and were chemically synthesized with a C-terminal amidation, to remove the C-terminal negative charge at neutral pH.

Although various routes have been suggested as the detailed mode of action of cationic, amphipathic helical AMPs, their interactions with the bacterial cell membranes are thought to be the key step [23,24,25,26,27]. In addition, the helical conformation is a fundamental requirement of our design concept to provide amphipathic properties. Thus, in order to ascertain that our model peptides could indeed fold into a helical structure in membrane environments, as assumed in their design, their far-UV CD spectra were measured in buffer solutions containing 50% TFE, 10 mM SDS micelles, and 2 mM DPC micelles, respectively. As a representative example, CD spectra of the W7 peptide are represented in Figure 1. As evidenced by the strong negative bands near 208 and 222 nm, all of the model peptides possessed an inherent propensity to adopt helical conformations in the three solutions tested. Helical contents ([α]) were predicted to vary between 64 and 86%, depending on individual peptides and solvents. A detailed inspection of the conformational behavior is discussed in later sections. The aqueous TFE mixture and detergent micelle solutions are generally employed as simple membrane-mimetic environments [36,37,38,39]. Although they might not exactly resemble biomembranes, the present results indicate that our model peptides could also adopt a helical conformation in actual membrane environments and thereby achieve amphipathic properties, as intended in the design step.

### 2.2. Variations in Activities

Table 1 summarizes the MIC values of our model peptide isomers against various bacterial species, including four Gram-positive, five Gram-negative and a multi-drug resistant strain. As normalized by GM (geometric mean of the MICs) values [21,40,41], the most potent activity was observed for the W10 (L_5_K_5_W^10^) peptide, of which the GM values were 1.2 and 1.7 μg/mL against Gram-positive and Gram-negative strains, respectively. In contrast, the W8 (L_5_K_5_W^8^) peptide showed the highest GM values, indicating the weakest activity: 3.4 and 4.6 μg/mL against Gram-positive and Gram-negative strains, respectively. Overall, however, it can be concluded that all of our model peptide isomers possessed a strong, broad-spectrum antibacterial activity with an average GM value of 2.7 ± 0.6 μg/mL (1.9 ± 0.4 μM), against the nine bacterial strains tested. In addition, all of the peptides were fairly active (MICs ≤ 16 μg/mL) against the MRSA-TK784 strain, which is not susceptible to β-lactam antibiotics and protein synthesis inhibitors and moderately resistant to vancomycin [21,42]. These results validate the effectiveness of our specific strategy for designing antimicrobial peptides and present the L_5_K_5_W^n^ peptide isomers as potent antibacterial agents.

**Table 1:** Antimicrobial activities of the L_5_K_5_W^n^ model peptides.

	W1 ^a^	W2	W3	W4	W5	W6	W7	W8	W9	W10	W11
Minimal inhibitory concentration (μg/mL)											
Gram-positive bacteria											
*B. subtilis*	2	2	2	2	2	2	2	4	2	1	2
*S. aureus*	2	2	2	2	2	2	2	2	2	1	2
*S. epidermis*	2	2	4	2	2	2	2	4	2	1	2
*M. luteus*	4	4	4	4	2	4	4	4	4	2	4
Gram-negative bacteria											
*E. coli*	4	2	4	4	2	2	2	4	2	2	4
*S. dysentariae*	2	1	2	2	2	2	2	4	2	1	2
*S. typhimorium*	2	2	4	4	2	4	4	4	2	1	4
*K. pneumoniae*	2	2	2	4	2	4	2	4	2	2	4
*P. aeruginosa*	8	8	8	4	8	8	8	8	4	4	4
GM (geometric mean of MICs)											
Gram (+) ^b^	2.4	2.4	2.8	2.4	2.0	2.8	2.4	3.4	2.4	1.2	2.4
Gram (−) ^c^	3.0	2.3	3.5	3.5	2.6	3.5	3.0	4.6	2.3	1.7	3.5
Gram (+,−) ^d^	2.7	2.3	3.2	2.9	2.3	3.2	2.7	4.0	2.3	1.5	2.9
Methicillin-Resistant *S. aureus*											
*MRSA-TK784*	8	8	8	16	8	4	16	16	8	4	16

^a^ Amino acid sequences are represented by the corresponding model numbers (refer to Figure 1); ^b^ against 4 Gram-positive strains tested; ^c^ against 5 Gram-negative strains tested; ^d^ against all of the 9 strains tested.

**Figure 2:**
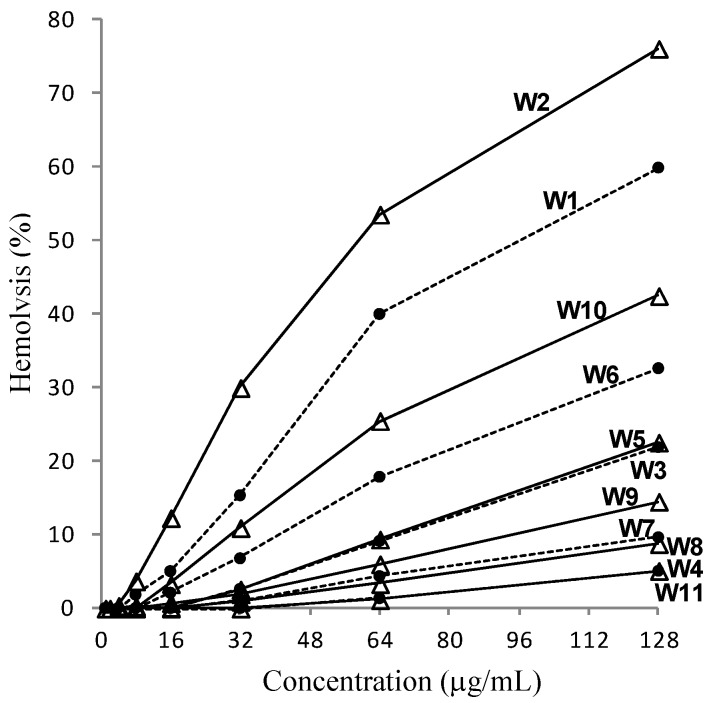
Hemolytic activities of the L_5_K_5_W^n^ model peptides. Human red blood cells were treated with individual peptides at various concentrations and hemolysis extents (%) are plotted along the peptide concentrations. Amino acid sequences are represented by the corresponding model numbers (refer to Figure 1).

To evaluate the potential use of the model peptides as therapeutic candidates, their hemolytic activities were examined (Figure 2). It was notable that hemolytic activities against human red blood cells varied dramatically between peptides, while their antibacterial activities were similar. Based on the % hemolysis values at a concentration of 128 μg/mL, the W2 peptide was the most hemolytic, whereas hemolysis by the W4 and W11 peptides was not significant. Finally, the balance between the antibacterial and hemolytic activities was simply approximated in Table 2 using the pseudo-therapeutic index (TI') values [21,40,41]. As discussed previously [21], the absolute value of TI' (TI' = MHC/GM), which is distinct from the typical therapeutic index (TI = LD_50_/ED_50_) for chemotherapy, cannot be used for a quantitative evaluation of safety. Nevertheless, it is reasonable to use the TI' values for a relative comparison of safety between peptides. In this respect, the W7 (L_5_K_5_W^7^) peptide, which showed the highest TI' values (53.8 for Gram-positive and 42.2 for Gram-negative bacteria), appears to be the best molecule for therapeutic development. In addition, when considering their potent antimicrobial activities even against antibiotic-resistant bacteria, the other peptides, even the W2 peptide, which had the lowest TI' values of 6.7–7.0, could be also regarded as potentially useful antibacterial agents. In particular, as all of our L_5_K_5_W^n^ peptides displayed negligible hemolytic activity in their MIC ranges (≤8 μg/mL), they are all promising templates for the development of effective therapeutics, through further optimization of amino acid sequences.

**Table 2:** Minimal hemolytic concentration (MHC) and pseudo-therapeutic index (TI') values of the L_5_K_5_W^n^ model peptides.

Peptides	MHC (μg/mL)		TI'		
			(+) ^a^	(−) ^b^	(+, −) ^c^
**L_5_K_5_W^1^ (W1)**	32		13.5	10.6	11.8
**L_5_K_5_W^2^ (W2)**	16		6.7	7.0	6.9
**L_5_K_5_W^3^ (W3)**	64		22.6	18.4	20.2
**L_5_K_5_W^4^ (W4)**	128		53.8	36.8	43.5
**L_5_K_5_W^5^ (W5)**	64		32.0	24.3	27.4
**L_5_K_5_W^6^ (W6)**	32		11.3	9.2	10.1
**L_5_K_5_W^7^ (W7)**	128		53.8	42.2	47.0
**L_5_K_5_W^8^ (W8)**	128		38.1	27.9	32.0
**L_5_K_5_W^9^ (W9)**	64		26.9	27.9	27.4
**L_5_K_5_W^10^ (W10)**	32		26.9	18.4	21.8
**L_5_K_5_W^11^ (W11)**	128		53.8	36.8	43.5

^a^ against four Gram-positive strains tested; ^b^ against five Gram-negative strains tested; ^c^ against all nine of the tested strains.

It is also noteworthy that some of our peptides possess a significant similarity in amino acid sequences with other known AMPs in the literature. For example, a great number of antimicrobial undecapeptides, including the best molecules BP76 and BP100, have been generated using a combinatorial peptide chemistry approach [43,44]. In particular, a striking similarity is observed between the W10 (L_5_K_5_W^10^) peptide, which showed the strongest antibacterial activity in the present study (Table 1), and the BP76 (sequence: KKLFKKILKFL-amide) and BP100 (sequence: KKLFKKILKYL-amide) peptides, which have potent bactericidal activity against diverse phytopathogenic bacteria. The L4 and L7 residues in the W10 peptide are replaced by other hydrophobic residues, F4 and I7, respectively, commonly in the BP76 and BP100 peptides. The tryptophan in the W10 peptide is substituted with similar aromatic amino acids, F10 and Y10, in the BP76 and BP100 peptides, respectively. Consequently, the MIC and the % hemolysis values of the W10 peptide were comparable to those of the BP76 and BP100 peptides. These comparisons also support that our model peptides would be promising for the development of useful antibiotics. The present work also included re-evaluation of the W6 (L_5_K_5_W^6^) peptide, which was derived from the previous preliminary study [22]. Based on the present results with more elaborate examination, the W6 peptide seems somewhat hemolytic. However, comparing to the W6 peptide, the other isomers, except for the W2 peptide, could be regarded as more improved molecules with higher TI' values.

### 2.3. Conformational Behavior

**Figure 3:**
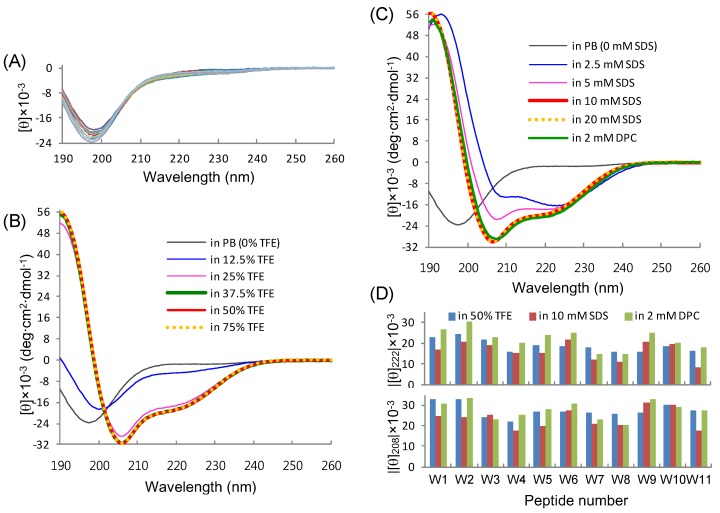
CD analysis of the L_5_K_5_W^n^ model peptides. (**A**) Far-UV CD spectra of individual peptides in 10 mM phosphate buffer at pH 6.5 are superimposed; (**B** and **C**) CD spectra of the W10 peptide in phosphate buffer containing various concentrations of TFE and detergents (SDS and DPC) are represented, respectively; (**D**) Absolute values of mean residue molar ellipticity at 222 nm ([θ]_222_; upper panel) and 208 nm ([θ]_208_; lower panel) are plotted for individual peptides in three different solutions.

In order to investigate the structure-activity relationships of our model peptides, their conformational preferences in various solvent compositions were inspected in detail by CD spectroscopy. Generally, the cationic, amphipathic helical AMPs adopt their helical conformations upon interaction with the membrane, while disordered in aqueous solution. Our L_5_K_5_W^n^ peptides were also mostly unstructured in aqueous buffer, as evidenced by the far-UV CD spectra in phosphate buffer (Figure 3A), which were characterized by a strong negative band below 200 nm. The [θ]_203_ values of the peptides, which are useful for validating peptide concentrations, were −16.3 ± 0.5 (standard deviation, 11 values; ±0.16 of standard error) × 10^3^ deg·cm^2^·dmol^−1^, which is in good agreement with the known value of −15.1 ± 1.6 for other peptides in the literature [39,45]. Upon the addition of a hydrophobic component (TFE) or detergent (SDS and DPC) micelles, the CD spectra changed into a shape that was characteristic of helix formation, which was based on the increase in signal intensity at 208 and 222 nm. As a representative example, the CD spectra of the W10 (L_5_K_5_W^10^) peptide, which showed the strongest antibacterial activity (Table 1), are represented in Figure 3B and Figure 3C. For other peptides, absolute values of [θ]_222_ and [θ]_208_ in three different solutions are presented for deducing the shape of each spectrum (Figure 3D). Spectral changes by adding TFE were saturated at either 37.5 or 50% TFE and most peptides showed an isodichroic point at around 202 or 203 nm, which supports a two-state equilibrium, probably between disordered and helical conformations [39,45,46,47]. In contrast, the isodichroic point was not apparent from the SDS-induced spectral changes of most peptides. Thus, at SDS concentrations below the critical micelle concentration (CMC) of approximately 8.7 mM [37,38,48], different conformations, such as coiled-coil oligomers [49,50], seem to also be present. However, the spectral changes for all of the peptides were saturated in the presence of SDS when the concentration was higher than the CMC, implying that a single, stabilized conformation was formed in the detergent micelle environment. Similarly, all of the peptides displayed stabilized helix conformations in 2 mM DPC micelles, which was above its CMC of approximately 1.5 mM [38,51]. However, the fine structures of spectra in the same solvent were not identical between peptides (Figure 3D) and the spectral shapes of the same peptide also varied depending on the environments (TFE solution, SDS micelles, and DPC micelles) without an apparent isodichroic point (Figure 1 and Figure 3). These results indicate that the functional, helical structures of the peptides would be altered in detailed conformations, depending on the structure and composition of the membranes where the peptides interacted.

### 2.4. Tryptophanyl Environments

**Figure 4:**
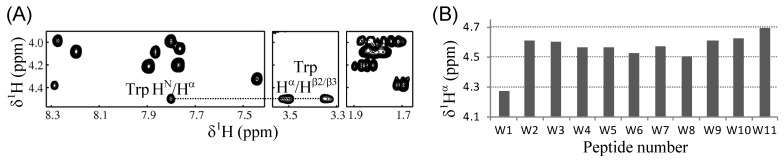
NMR analysis of the L_5_K_5_W^n^ model peptides. (**A**) Selected regions from the 2D-COSY spectrum of the W8 peptide in 50% TFE solution are aligned to present the ^1^H^α^-^1^H^β^^2/β^^3^ connectivity of the tryptophan residue (dotted line); (**B**) Tryptophan ^1^H^α^ chemical shifts of individual peptides in the 50% TFE solution are plotted.

In our peptide design, the single tryptophan was always introduced at a critical amphipathic interface. The tryptophan at this specific position has been proposed to play an important role in membrane interaction of the peptides, by stabilizing the amphipathic properties of the helices and/or by anchoring the peptides into membranes [28,29,30]. Thus, in order to assess the role of the tryptophan in our peptides, we first checked whether the tryptophan was involved in the helical region of individual peptides, by NMR spectroscopy. It is well known that the alpha proton chemical shifts (δ^1^H^α^) of a protein are highly sensitive to its secondary structure and that an upfield shift of a δ^1^H^α^ from its random-coil reference value indicates a helical tendency of the corresponding residue [52]. As an example of NMR assignments, Figure 4A illustrates the ^1^H^N^-^1^H^α^-^1^H^β^^2/β^^3^ connectivities observed in the 2D-COSY spectrum of the W8 (L_5_K_5_W^8^) peptide in the 50% TFE solution, which possessed the weakest antibacterial activity (Table 1). Based on the distinguished δ^1^H^β^ value of tryptophan (3.27~3.63 ppm for the present peptides) and its three-bond coupling to the ^1^H^α^, the tryptophan δ^1^H^α^ of individual peptides could be easily assigned. As shown in Figure 4B, the tryptophan δ^1^H^α^ values of most peptides were significantly (≥0.1 ppm) lower than its random-coil reference value, 4.7 ppm [52]. These results imply that the tryptophans in our model peptides were involved in the helical region, likely stabilizing the amphipathic properties of the helices, as previously observed for other similar peptides [30]. The only exception was the W11 peptide, of which tryptophan δ^1^H^α^ was close to the random-coil value. This was attributable to the fact that W11 peptide contained the tryptophan at the C-terminus, which would promote the flexibility of the residue.

**Figure 5:**
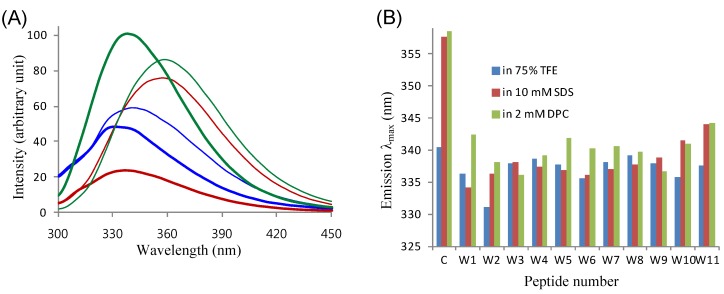
Intrinsic tryptophan fluorescence of the L_5_K_5_W^n^ model peptides. (**A**) Fluorescence emission spectra of NATA (thin lines) and the W2 peptide (thick lines), in 75% TFE (blue), 10 mM SDS (red) and 2 mM DPC (green) solutions are represented; (**B**) Emission λ_max_ in each solution is plotted for individual peptides and NATA (denoted by ‘c’, control).

The microenvironments of tryptophans were further investigated by fluorescence spectroscopy (Figure 5). Fluorescence emissions of our peptides originate solely from the single tryptophans. Thus, we used *N*-acetyl-L-tryptophanamide (NATA) as a control material for fluorescence measurements, as it is an indole derivative that closely mimics a tryptophan residue involved in peptide bonds [28,29]. Figure 5A shows the fluorescence emission spectra of NATA and the W2 (L_5_K_5_W^2^) peptide, which was the most hemolytic among our peptides, in the three different environments where the peptides were stably folded into helical conformations. For the other peptides, the emission λ_max_ values are presented in Figure 5B. The λ_max_ values of our peptides in the aqueous TFE solution were lower than that of NATA, by 3.5 ± 2.2 nm. Since the aqueous TFE solution is an isotropic environment, the difference in λ_max_ between NATA and a peptide (Δλ_max_ = λ_max,peptide_ − λ_max,NATA_) is attributable to the local conformation of tryptophan, which is inevitably governed by the secondary structure of the peptide. Therefore, it can be postulated again that the tryptophan residues in most of the peptides were likely involved in helix formation, as were observed in the NMR results. Then, the Δλ_max_ was further enlarged in detergent micelle solutions, with −19.6 ± 2.7 in SDS and −18.6 ± 2.4 nm in DPC micelles. As detergent micelle solutions produce anisotropic environments, the profound blue shift of λ_max,peptide_ from λ_max,NATA_ was mainly attributed to the position of tryptophan, as well as the conformational effect. Thus, the results imply that the tryptophan residues of our peptides were efficiently inserted into the hydrophobic interior of micelles, while NATA resided outside or on the hydrophilic surface of micelles. Taken together and consistent with our previous observations [28,29,30], these results suggest that the tryptophans in our model peptides likely play a role anchoring the peptides into membranes to drive their membrane permeation.

### 2.5. Structure-Activity Relationships

Structure-activity relationships of cationic, amphipathic helical AMPs are generally elucidated in terms of several physico-chemical properties and structural parameters influencing activity. Those include the peptide length or number of amino acids, net positive charge, polar angle, mean residue hydrophobicity (*H*) or retention time on reverse-phase HPLC (*t_R_*), amphipathicity or hydrophobic moment (*μH*), and helicity or helical content ([α]) [23,24,53,54,55]. As many of these parameters are coupled to one another, the activity appears usually as a complicated, cooperative resultant of the interactive parameters. However, our L_5_K_5_W^n^ peptides enable a simplified inspection of structure-activity relationships, because they are related as isomers with conserved properties. For example, peptide size (11 amino acids with molecular weight of 1410 Da) and net positive charges (+7 at neutral pH) are identical. Likewise, theoretical *H* (−0.22 on the Eisenberg scale [21,56]) is the same and practical *t_R_* (20.8 ± 1.1 min on a C18 column; data not shown) was not significantly variable. Polar angles (either 120° or 140° in the helical wheel diagram; refer to Figure 1) and *μH* values (0.51 ± 0.015 on the Eisenberg scale), expected as ideal helices, are also similar. Accordingly, the only variable parameter of our peptides is the helicity, which can be estimated from CD spectra. Then, additional structural parameters for our peptides can be derived from fluorescence emission spectra, which reflect the local conformation around tryptophan. In our peptide design, the tryptophans were specifically incorporated in expectation of a structural role for conferring activity. 

**Figure 6:**
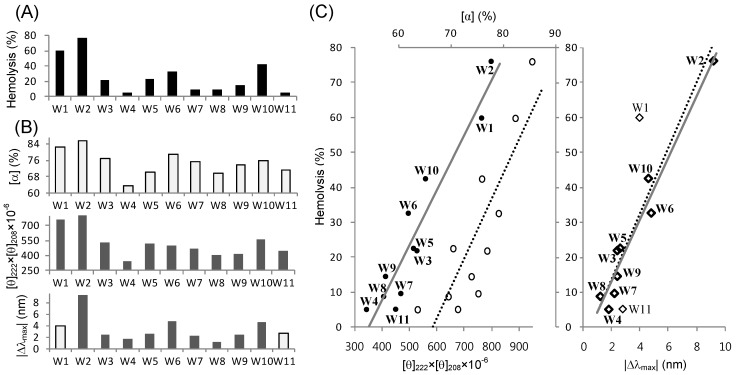
Relationships between hemolytic activity and structural parameters of the L_5_K_5_W^n^ model peptides. (**A**) Hemolysis extents (%) by individual peptides at a 128 μg/mL concentration were extracted from Figure 2; (**B**) Structural and spectroscopic parameters of individual peptides in 50% (for CD) or 75% (for fluorescence) TFE solutions. Predicted helical contents ([α]; upper panel) and the [θ]_222_ × [θ]_208_ values (middle panel) were derived from the CD data in Figure 3. Differences in λ_max_ (|Δλ_max_|; bottom panel) between peptides and NATA were derived from fluorescence emission data in Figure 5. Light gray bars correspond to the light symbols in **C**; (**C**) Linear correlation between hemolytic activity and structural parameters in 50% (for CD) or 75% (for fluorescence) TFE solutions. The hemolysis % is plotted along the [θ]_222_ × [θ]_208_ (filled circles with bottom coordinate; left panel) and [α] (empty circles with upper coordinate; middle panel), respectively, with the corresponding regression lines determined by linear least square fitting. The coefficients of determination R^2^ were 0.9022 (solid line) and 0.7321 (dotted line), respectively. The hemolysis % is also plotted along the |Δλ_max_| (diamonds; right panel). The linear regression lines were derived using all 11 data points (dotted line; R^2^ = 0.7776) and 9 selected data points (solid line with bold diamond symbols; R^2^ = 0.9518), respectively.

We examined the relationships between the activities of our peptides and their structural parameters derived from CD and fluorescence spectra. Unfortunately, however, we could not identify any apparent relationship between the antibacterial activity and a single structural parameter, because the variation in antibacterial activity between peptides was too small to statistically analyze the correlation. Unlike the MIC values, which are labile depending on the bacterial species tested, the obvious variation in hemolysis % at a peptide concentration of 128 μg/mL allowed us to identify good correlations between hemolytic activity and structural parameters. First, the hemolysis % values (Figure 6A) of the 11 peptide isomers and their helical contents (Figure 6B) in 50% TFE solution showed similar dispersion patterns. As mentioned above, 50% TFE is the minimal concentration where the coil-to-helix transitions of all of our peptides are completed. Least square regression analysis of the two data sets (hemolysis *versus* helical contents) showed an appreciably linear correlation with an R^2^ value of 0.7321 (Figure 6C). The linearity was not apparent or significantly reduced at TFE concentrations below 50% (data not shown), where some peptides are not yet finally stabilized in a helical conformation. The helical contents can be altered by programs or algorithms used to predict the secondary structure from CD data. However, when we also simply used the product values of [θ]_222_ and [θ]_208_ ([θ]_222_ × [θ]_208_), instead of the predicted helical contents, the regression line showed increased goodness of linear fit, with an R^2^ value of 0.9022. The wavelengths 222 and 208 nm are the indicating points of helix formation [48,57]. Thus, these two regression results clearly suggest that the TFE-induced helicity of our L_5_K_5_W^n^ peptides was well correlated with their hemolytic activity.

Figure 6C also shows a linear (R^2^ = 0.7776) correlation between hemolytic activity and the fluorescence emission λ_max_ in the 75% TFE solution. Given that the W1 and W11 peptides, which have a tryptophan at the N- and C-terminus, respectively, are distinguished from the other peptides, their fluorescence would differ from others and may not correctly reflect the local conformation around the tryptophan. When we analyzed the data with just nine peptides, excluding the W1 and W11 peptides, the regression R^2^ reached 0.9518, indicating a remarkably linear correlation. These results suggest that the TFE-induced local conformation around the single tryptophan was strongly related to the hemolytic potency of our L_5_K_5_W^n^ model peptides. This, in turn, also suggests that the tryptophans in our model peptides would play prominent roles in dictating their activity.

In summary, the present results showed that structural parameters of our model peptides, represented as helicity and tryptophan fluorescence, in TFE solutions could be a useful probe to predict the relative potency of hemolytic activity. However, in detergent (SDS and DPC) micelles, no significant correlation between the hemolytic activity and the structural parameters ([α], [θ]_222_ × [θ]_208_, and |Δλ_max_|) was observed (data not shown). It is no guarantee that the TFE solution is a better membrane-mimetic environment than the detergent micelles, because none of the media can exactly resemble the diverse biomembranes. However, restricted to the present model peptides, it can be postulated that the TFE-induced helical adoption could more closely resemble the active conformations formed upon interaction with the erythrocyte membrane. Nonetheless, the functional structures in bacterial membranes can be different from the TFE-induced conformations. As mentioned above, the results presented in this study indicated that the detailed conformations of our model peptides varied in different environments, although they similarly adopted helical structures. Thus, the conformational flexibility of the peptides would be critically correlated with their activity and selectivity.

As mentioned above, the present model peptides are expected to have conserved physico-chemical properties, as they are related as isomers. However, despite overall similarity, detailed profiles of hydrophobicity and amphipathicity can vary along the helix axis. For example, the W4 and W8 peptides possess two hydrophobic leucines at the N-terminus and two hydrophilic lysines at the C-terminus, whereas the W6 peptide shows an opposite pattern. The anisotropic contributions of hydrophobic/hydrophilic residues, combined with the different positions of tryptophans, may result in different helical propensity and different tilt of individual peptides when bound into the membranes. Thus, the detailed structures and membrane orientations of the peptides would be worthy of further investigation to clearly elucidate their structure-activity relationships.

## 3. Experimental

### 3.1. Peptide Preparation

Chemically synthesized model peptides were purchased as dry powders from the peptide manufacturing company AnyGen (Kwangju, Korea; http://www.anygen.com). The purity and the correct mass of the product peptides were determined by HPLC and mass spectrometry, respectively. Concentration of each peptide dissolved in its designated solvent was determined spectrophotometrically, using the known value of molar absorptivity for tryptophan (5,500 M^−1^cm^−1^ at 280 nm), since the present peptides commonly possess single tryptophan residues.

### 3.2. Circular Dichroism (CD) Spectroscopy

Far-UV CD spectra of 45 μM peptides dissolved in 10 mM phosphate buffer (PB) containing 0–75% TFE, 0.625–20 (two-fold serial dilutions) mM SDS micelles and 2 mM DPC micelles, respectively, were measured on a Applied Photophysics Chirascan CD spectrometer, using a 0.2 cm path-length cell, with a 1 nm bandwidth and a 0.5 nm step resolution, at 20 °C. Three individual scans taken from 260 to 190 nm were summed and averaged, followed by subtraction of the solvent CD signal. Finally, the CD intensity at a wavelength λ was normalized as the mean residue molar ellipticity, [θ]_λ_ (deg·cm^2^·dmol^−1^) [28]. The helical contents were deduced based on curve fitting using the CDNN 2.1 program [58].

### 3.3. Nuclear Magnetic Resonance (NMR) Spectroscopy

Samples for NMR measurements contained 1.5 mM peptide in TFE-*d_3_*/H_2_O (1:1, v/v) at pH 5.0. The conventional 2D COSY spectrum of each peptide was measured at 298 K, using Bruker Avance 500 MHz and 800 MHz spectrometers equipped with a cryoprobe. The ^1^H chemical shifts were referenced directly to the methyl signals of DSS (sodium 4,4,-dimethyl-4-silapentane-1-sulfonate).

### 3.4. Fluorescence Spectroscopy

Fluorescence emissions of 5 μM peptides and NATA dissolved in designated solvents were monitored at 20 °C, on a JASCO FP-6500 spectrofluorometer, using a 10 mm quartz cell. An excitation wavelength of 280 nm was used with a 5 nm excitation bandwidth. The fluorescence emission was recorded at every 0.2 nm, with a 5 nm emission bandwidth and a 0.5 s response time. Three individual scans taken from 300 to 450 nm were added and averaged.

### 3.5. Antimicrobial Assay

The antimicrobial activity of each peptide was determined against four strains of Gram-positive bacteria (*Bacillus subtilis* ATCC 6633, *Staphylococcus aureus* ATCC 6538p, *Staphylococcus epidermis* ATCC 12228, and *Micrococcus luteus* ATCC 10240) and five strains of Gram-negative bacteria (*Escherichia coli* ATCC 25922, *Shigella dysentariae* ATCC 9752, *Salmonella typhomurium* ATCC 14028, *Klebsiella pneumoniae* ATCC 10031, and *Pseudomonas aeruginosa* ATCC 27853). In addition, a methicillin-resistant *Staphylococcus aureus* strain, MRSA-TK784 [21,42], was employed as a multi-drug resistant bacterium. Antimicrobial susceptibility was measured as the minimal inhibitory concentration (MIC), which was determined by the standard broth microdilution method, as described previously [21]. Briefly, the MIC was defined as the lowest peptide concentration that completely inhibits cell growth from cell cultures incubated in the presence of various concentrations (1–128 μg/mL, two-fold serial dilutions) of peptides. The tests were performed in triplicate, and the MIC values that were reproduced twice or three times in the three independent measurements were recorded.

### 3.6. Hemolytic Assay

The hemolytic assay was performed as described previously [21]. In brief, suspensions of human red blood cells (10% v/v in PBS) were treated for 1 h with various concentrations (1–128 μg/mL, two-fold serial dilutions) of the peptides. The absorbance of the supernatant was then measured at 550 nm. The relative value to 100% hemolysis of human red blood cells treated with 0.2% Triton X-100 was defined as the percentage of hemolysis. The tests were performed using triplicate samples, and the average values of the three independent measurements were recorded. The minimal hemolytic concentration (MHC) value was determined as the lowest peptide concentration that produces more than 5% hemolysis [21,40,41,53].

## 4. Conclusions

In this study, 11 L_5_K_5_W^n^ undecapeptide isomers were generated under our specific rule to design amphipathic helical peptides, and all of them exhibited potent antibacterial activity. Then, spectroscopic parameters in aqueous TFE solutions could be suggested as simple probes correlating their structural properties with remarkable variations in hemolytic activities. Although a huge number of AMPs are available, reducing toxicity is one of the most important and prerequisite processes for their practical development as therapeutic agents. Particularly, hemolysis test is often employed as the first step to evaluate toxicity of AMPs. Thus, we expect that the present results will provide a well-designed research template for a rational, systematic approach for the *de novo* design of AMPs. 

In conclusion, this study established a useful method to design small, potent antimicrobial peptides with a simple amino acid composition and to characterize their structure-activity relationships. Additionally, we expect that the present structural results will contribute to further optimization of the L_5_K_5_W^n^ model peptides as promising templates for novel antibiotic development.
